# Studies of human pancreatic elastase treatment of rabbit and human vein rings to predict human therapeutic doses

**DOI:** 10.1002/prp2.229

**Published:** 2016-04-02

**Authors:** Steven K. Burke, David Bunton, Karen Bingham, Emma Moss, Kimberly S. Bland, Barry Starcher, Marco D. Wong, F. Nicholas Franano

**Affiliations:** ^1^ Proteon TherapeuticsWalthamMassachusetts; ^2^ Biopta, Ltd.GlasgowUnited Kingdom; ^3^ The University of Texas Health Science CenterTylerTexas; ^4^ Present address: Stowers Institute for Medical ResearchKansas CityMO; ^5^ Present address: FlowForward MedicalOlatheKS

**Keywords:** CELA1, chymotrypsin‐like elastase family member 1, elastase, elastin, hemodialysis access, PRT‐201, vein, vonapanitase

## Abstract

Vascular tissue contains abundant elastic fibers that contribute to vessel elasticity. Vonapanitase (formerly PRT‐201) is a recombinant human chymotrypsin‐like elastase family member 1 (CELA1) shown to cleave the elastin component of elastic fibers, resulting in increased vessel diameter. The purpose of these current studies was to determine vein diameter, wall thickness, elastin content, and vonapanitase potency in veins used in a model of arteriovenous fistula (AVF) and in patients undergoing AVF creation for hemodialysis access to guide dose selection for human trials. Rabbit linguofacial, maxillary, and external jugular veins, and human basilic and upper and lower arm cephalic veins were dissected postmortem and sectioned into 2 mm length rings. Rings were incubated in vonapanitase at 37°C at varying concentrations and times. Elastin content was estimated histologically and by quantifying desmosine, a protein cross‐link unique to elastin. Rabbit veins were substantially thinner and contained less elastin than human veins. In human veins, elastin content was greatest in basilic and least in lower arm cephalic. Vonapanitase removed elastin in a time‐ and concentration‐dependent manner in all vein types. A lower concentration of vonapanitase was required to remove elastin from rabbit relative to human veins. In summary, vonapanitase reduced the elastin content of rabbit and human veins but did so at a lower concentration in the rabbit veins. Rabbit models may overestimate the potency of vonapanitase in humans. These results indicate that human dose selection should be guided by human vein ring experiments.

**Abbreviations**
AVF: arteriovenous fistula
AVG: arteriovenous graft
CELA1: chymotrypsin‐like elastase family member 1
CKD: chronic kidney disease
MMA: methyl methacrylate
PBS: phosphate‐buffered saline
VVG: Verhoeff–Van Gieson

## Introduction

Vonapanitase is a 26‐kDa recombinant human chymotrypsin‐like elastase family member 1 (CELA1) (Kawashima et al. 1992). Vonapanitase fragments elastin and removes it from treated tissues, including blood vessels. Removal of elastin with CELA1 has been shown to increase artery and vein diameter (Dobrin et al. 1984; Kitoh et al. 1993), increase blood flow (Burke et al. 2008), relieve vasospasm (Chew et al. 2004), and reduce the formation of neointimal hyperplasia following vascular injury (Amabile et al. 2002; Wong et al. 2002). Vonapanitase has been studied in human arteriovenous fistula (AVF) and arteriovenous graft (AVG) clinical trials (www.clinicaltrials.gov – NCT00679991, NCT01305824, and NCT01001351), in which vonapanitase is applied topically to surgically exposed outflow veins immediately after AVF/AVG creation to assess the effects on maturation and patency. An AVF or AVG is essential for persons with chronic kidney disease to receive life‐sustaining hemodialysis.

The purposes of these current studies were to determine vein diameter, wall thickness, vein elastin content, and vonapanitase potency for elastin removal in veins employed in a common animal model of AVF and veins used in patients undergoing AVF creation (Jones et al. 1994; Tronc et al. 1996; Masuda et al. 1999). In rabbit AVF models, it has been shown that elastin fiber fragmentation is an essential component of vascular remodeling and necessary for AVF maturation (Jones et al. 1994; Masuda et al. 1999; Tronc et al. 2000; Sho et al. 2002). Results of the current studies were used to determine the optimal vonapanitase doses for AVF and AVG clinical trials.

## Materials and Methods

### Postmortem vein harvest

Rabbit veins were obtained from four New Zealand white rabbits (12‐week‐old males, weighing between 2.2 and 2.4 kg). One rabbit was used for a pilot study and three rabbits were used for the main study. Each rabbit was sacrificed and left intact overnight at 2–8°C before vein dissection. In rabbits, the facial and lingual veins join to form the linguofacial vein which is subsequently joined by the maxillary then vertebral veins to form the external jugular vein. The dissected linguofacial veins from the right and left sides of the neck were cut into rings approximately 2 mm in length and mixed to create a pool of rings. In the full study, the dissected linguofacial, maxillary, and external jugular veins from the right and left sides of the neck were cut into rings and pooled. The animal portion of the study was carried out in accordance with the Guide for the Care and Use of Laboratory Animals, as adopted and promulgated by the United States National Institutes of Health, and was approved by the Institution's Animal Care and Use Committee.

The human veins were obtained by National Disease Research Interchange (NDRI, Philadelphia, PA) from deceased donors who donated their bodies to science. The protocol was approved by the National Institutes of Health (grant number U42 RR006042). Each donor or a family member signed an informed consent document prior to vein harvest. The basilic and cephalic arm veins of five donors were harvested within 24 h of death and transported in a physiological saline solution on ice to Biopta Ltd., Glasgow, where upper arm basilic, upper arm cephalic, and lower arm cephalic veins were sectioned into rings approximately 2 mm in length.

### Treatment

Individual rings were randomly selected from the pooled rings and placed in 1.5 mL microcentrifuge tubes containing 0.5 mL of phosphate‐buffered saline (PBS), pH 7.0, with vonapanitase or without vonapanitase (control). The microcentrifuge tubes were placed in a rack in a water bath maintained at 37°C. In the rabbit, pilot study rings were exposed to vonapanitase 0.5 mg/mL for 10, 15, 20, or 30 min and 0.1, 0.5, and 1.0 mg/mL for 15 min. Three rings were studied per condition, two rings were used for desmosine determination and one ring was used for histology. In the main rabbit study, rings were exposed to vonapanitase 0.25 mg/mL for 5, 15, 30, and 60 min and at 0.1, 0.25, 0.5, and 1.0 mg/mL for 15 min. Four rings were studied per condition, three rings were used for desmosine determination and one ring was used for histology.

In a pilot human study, lower arm cephalic vein rings from one donor and upper arm basilic vein rings from another donor were exposed to vonapanitase 0.5 mg/mL for 10, 15, 20, or 30 min and 0.1, 0.5, and 1.0 mg/mL for 15 min. In the main human study, veins from three donors were used. The left and right basilic, lower arm cephalic, and upper arm cephalic veins were harvested and each vein type from each arm was studied separately. The rings were exposed to vonapanitase 0.5 mg/mL for 5, 15, 30, and 60 min and 0.1, 0.5, 1.0, and 2.0 mg/mL for 15 min. After treatment, the rings were rinsed with PBS and then placed into 10% neutral buffered formalin for fixation. Three rings from each treated vein from each arm were used for desmosine determination and one ring was retained for histology.

### Measurements

For desmosine (i.e., elastin) determination, vein rings were hydrolyzed with hydrochloric acid solution, 6N, and then dried in a speed vacuum and suspended with water to a protein concentration of 0.5 mg/mL using a ninhydrin‐based assay (Starcher 2001). Desmosine is an amino acid cross‐link derived from four lysines that is unique to elastin fibers. Desmosine was measured by desmosine radioimmunoassay and content was expressed as pmol desmosine/mg protein (Starcher and Mecham 1981). For histology, vein rings were embedded in methyl methacrylate (MMA) blocks, sectioned and stained with Verhoeff–Van Gieson (VVG) stain at Charles River Pathology Associates, Frederick, MD. With VVG staining, elastin in the elastic fibers appears blue‐black. Slides of control rings were scanned with an Aperio scanner and the images underwent histomorphometry for measurement of lumen diameter, vein diameter, and wall thickness using Image‐Pro Plus software version 6.2 (MediaCybernetics, Bethesda, MD). The image was calibrated and the circumferences of the vein and the vein lumen were traced. These data were exported into Microsoft Excel (Redmond, WA) and the vein and lumen diameters were calculated as circumference/3.14. The vein wall thickness was calculated by subtracting the lumen diameter from the vein diameter.

### Statistics

For vein diameter, lumen diameter, wall thickness, and desmosine, groups were compared using unpaired *t* tests using Microsoft Excel and GraphPad Prism (La Jolla, CA). No adjustments were made to the level of significance to account for multiple comparisons.

## Results

### Pilot studies

In the rabbit pilot study, vonapanitase at a concentration of 0.5 mg/mL reduced the desmosine content by averages of 86%, 76%, 97%, and 69% after 10, 15, 20, and 30 min, respectively. Vonapanitase at a concentration of 0.1 mg/mL for 15 min did not reduce desmosine content, whereas treatment with 0.5 and 1 mg/mL for 15 min reduced the desmosine content by 76% and 72%. Since 0.1 mg/mL did not reduce desmosine content and 0.5 mg/mL removed almost all the desmosine after 10 min, an intermediate concentration of 0.25 mg/mL was selected for the main study time‐course. In the human pilot study of lower arm cephalic and upper arm basilic veins (combined *n* = 4 per condition), treatment with vonapanitase at a concentration of 0.1 mg/mL for 15 min did not reduce desmosine content (+17% vs. vehicle treatment), whereas treatment with 0.5 and 1 mg/mL for 15 min vonapanitase reduced the desmosine content by averages of 40% and 70%. Vonapanitase at 0.5 mg/mL reduced the desmosine content by 42%, 40%, 51%, and 62% after 10, 15, 20, and 30 min, respectively. Since 0.1 mg/mL did not reduce desmosine content and 1.0 mg/mL removed almost all the desmosine after 15 min, an intermediate concentration of 0.5 mg/mL was selected for the main study time course.

### Main study

**Figure 1:**
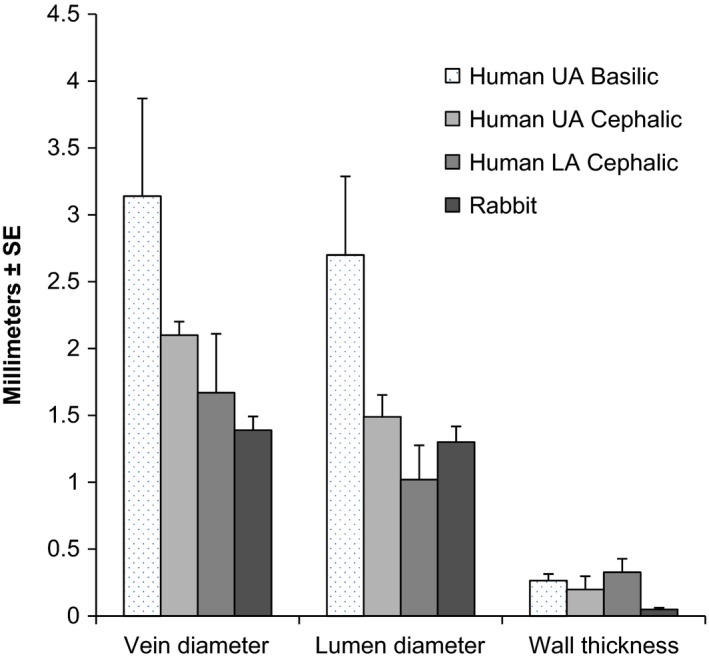
Histomorphometry of PBS‐treated vein rings for basilic vein, upper arm cephalic vein, and lower arm cephalic vein from human and a mix of linguofacial, maxillary, and external jugular vein from rabbits (*n* = 6 for each). UA, upper arm; LA, lower arm.

Figure 1 summarizes the histomorphometric measurements for the control veins. The largest vein and lumen diameters were found in the basilic vein, while the smallest diameters were found in the lower arm cephalic veins and rabbit veins. Vein wall thickness was similar for all vein types with the exception of the rabbit veins, which were notably thinner than the human veins. The average wall thickness for human veins was 0.28 ± 0.19 mm. In contrast, the average wall thickness for the rabbit veins was 0.05 ± 0.03 mm, or approximately 20% of the thickness of the average human vein. Average rabbit vein diameter was significantly smaller (*P* < 0.05) compared with basilic and upper arm cephalic but not lower arm cephalic. Average rabbit lumen diameter was significantly smaller than basilic only. Average rabbit vein wall thickness was significantly smaller than basilic and lower arm cephalic but not upper arm cephalic.

**Figure 2:**
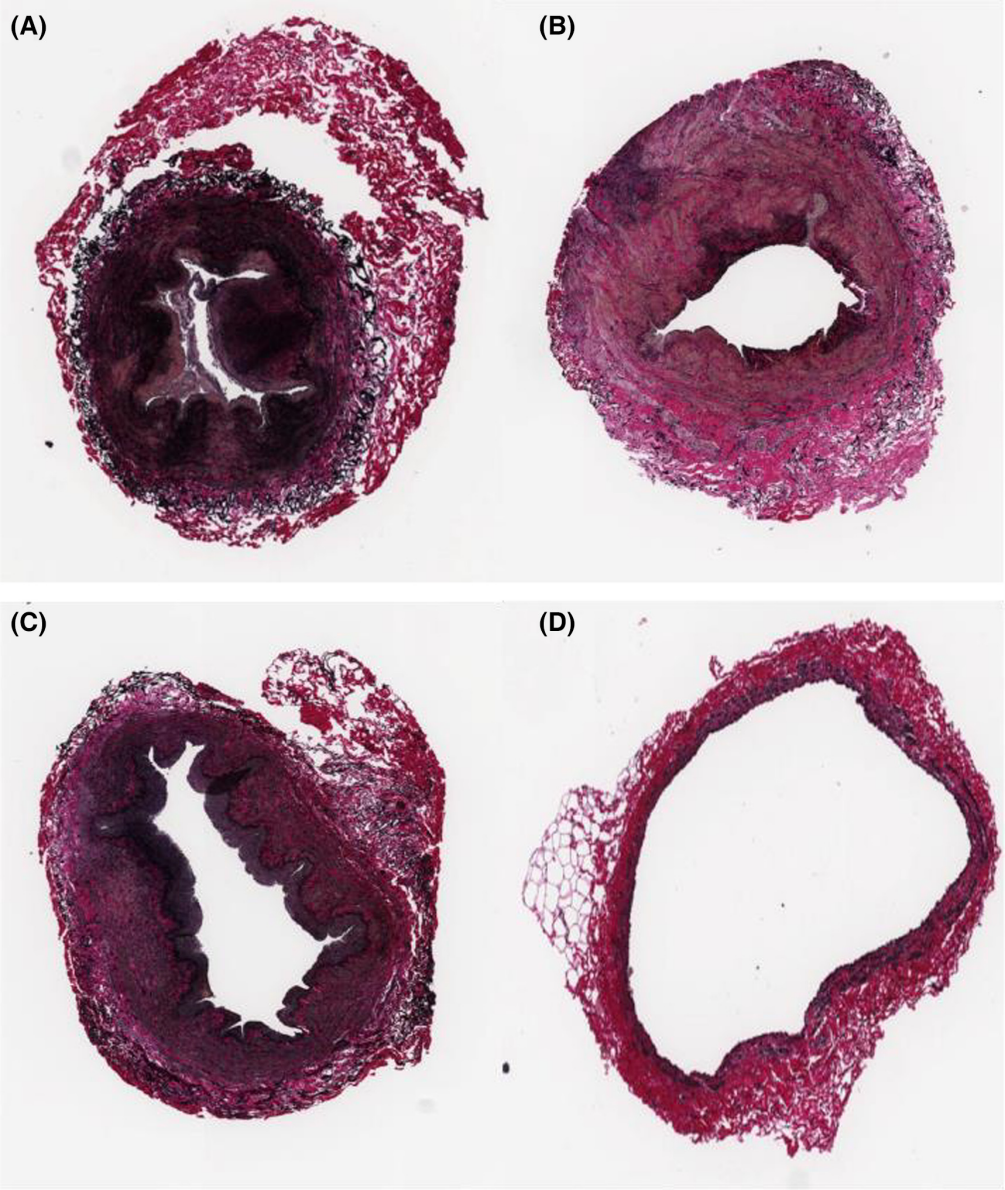
Histology of PBS‐treated vein rings – human basilic (A), upper arm cephalic (B), lower arm cephalic (C), and rabbit linguofacial (D). VVG stain at 20× magnification showing blue‐black elastic fibers.

**Figure 3:**
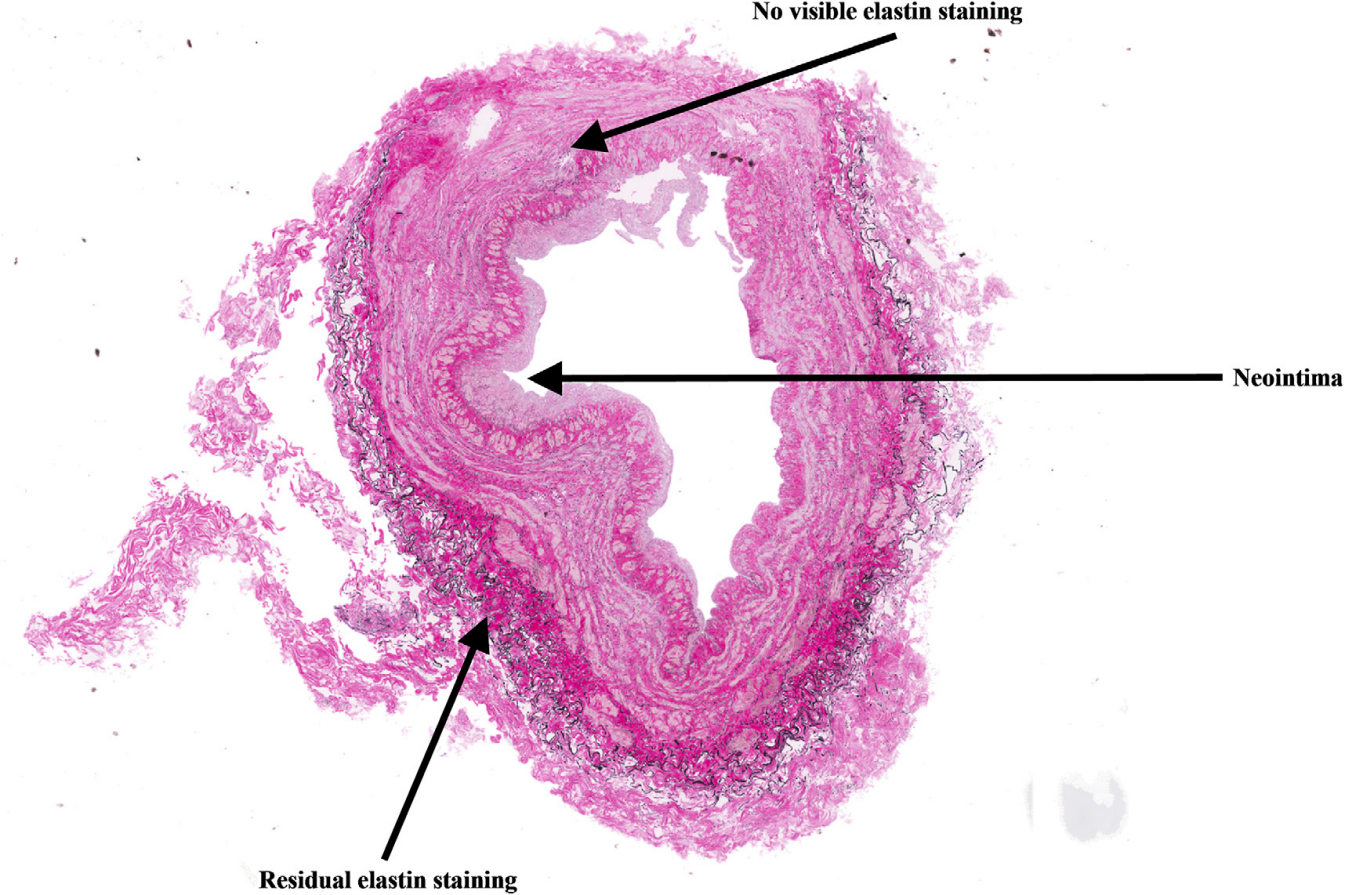
Lower arm cephalic vein treated with vonapanitase 0.1 mg/mL for 15 min (VVG stain at 20 × magnification).

Figure 2 shows representative images of control vein rings from one human donor and a rabbit linguofacial vein. Figure 3 shows a representative image of a lower arm cephalic vein treated with vonapanitase 0.1 mg/mL for 15 min. The majority of the vein wall is devoid of elastic fiber staining, with some spared areas, especially in the adventitia (outermost layer of the blood vessel).

**Figure 4:**
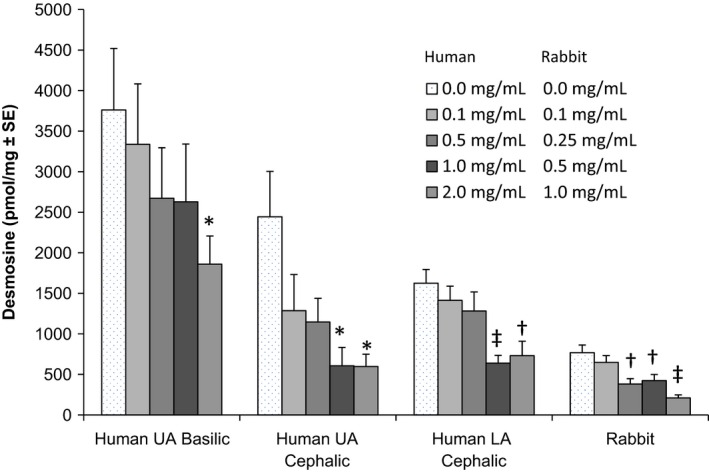
Desmosine (elastin) content of vein rings by vonapanitase concentration following a 15‐min treatment time. For human basilic and lower arm cephalic veins, *n* = 12 replicates (duplicate rings from the left and right arms of three donors) and for human upper arm cephalic, *n* = 8 replicates (duplicate rings from the right and left arms of two donors). For rabbit veins, *n* = 18 replicates (18 rings from a pool created from three rabbits). *P < 0.05, ^†^ *P* < 0.01, ^‡^ *P* < 0.001.

**Figure 5:**
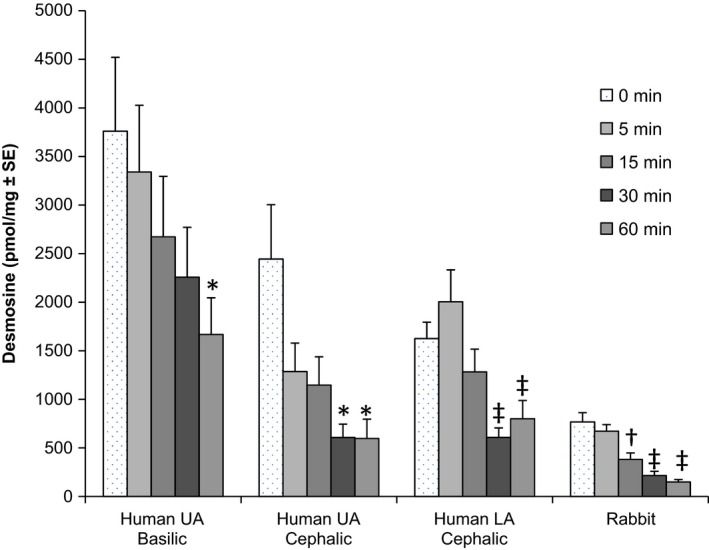
Desmosine (elastin) content by treatment time at a vonapanitase concentration of 0.5 mg/mL for human vein and 0.25 mg/mL for rabbit vein. For human basilic and lower arm cephalic veins, *n* = 12 replicates per time point (duplicate rings from the left and right arms of three donors) and for human upper arm cephalic, *n* = 8 replicates per time point (duplicate rings from the right and left arms of two donors). For rabbit veins, *n* = 18 replicates per time point (18 rings from a pool created from three rabbits). *P < 0.05, ^†^ *P* < 0.01, ^‡^ *P* < 0.001.

Figure 4 displays the relationship between vonapanitase concentration and desmosine (i.e., elastin) content following a 15‐min treatment time. In the control vein (0 mg/mL), desmosine content varied by vein location, even when normalized for protein content. The basilic vein had the highest desmosine content per mg of protein and the rabbit vein had the lowest. In vonapanitase‐treated rings, desmosine content was reduced in a concentration‐dependent manner from all locations. The 1 mg/mL concentration reduced the average desmosine content by 30%, 75%, 61%, and 72% for the basilic, upper arm cephalic, lower arm cephalic, and rabbit vein rings, respectively. Figure 5 displays the relationship between time and desmosine content following treatment with vonapanitase at a concentration of 0.5 mg/mL for human veins and 0.25 mg/mL for rabbit vein. In vonapanitase‐treated rings, desmosine content was reduced in a time‐dependent manner in vein from all locations.

## Discussion and Conclusion

Human recombinant CELA1 is under development as a treatment to promote the success of AVF surgery. Fragmentation of elastin, a process accelerated by the application of vonapanitase, is an early and essential component of AVF maturation (Jones et al. 1994; Masuda et al. 1999; Tronc et al. 2000; Sho et al. 2002). Elastase administered in doses resulting in partial elastin fragmentation has also been shown to reduce the formation of neointimal hyperplasia following vascular injury (Amabile et al. 2002; Wong et al. 2002), providing a potential mechanism whereby vonapanitase could promote AVF maturation or prevent AVF patency loss.

The purpose of this work was to better define the vonapanitase dose–response curve for elastin removal from veins that are commonly used to create an AVF for hemodialysis. As shown here, vonapanitase removed elastin from veins in a concentration‐ and time‐related manner, as assessed by both desmosine content and histological staining. These two methods of detecting elastin are complementary in that desmosine content allows for a quantitative assessment of elastin removal whereas histology allows for a visualization of the pattern of elastin removal (Masuda, 1999).

These studies provide evidence that vonapanitase treatment results in elastin removal in all vein types studied including those commonly used in rabbit AVF models and the human veins used in the creation of AVFs for hemodialysis. Although there are differences in species, vessel diameters, wall thickness, and elastin content among the veins studied, elastin removal with vonapanitase treatment was generally similar when expressed as a percentage reduction, with the exception being that of the basilic vein where at a dose of 1 mg/mL only 30% of elastin (desmosine) was removed after 15 min versus 61–72% for the other vein types.

One limitation of the current study is that the veins were not obtained from patients with chronic kidney disease (CKD). Patients with CKD suffer from high rates of cardiovascular disease and may have significant venous pathology (Lee et al. 2011, 2012). A second limitation of the model is that the veins were treated ex vivo and are not under pressure‐induced stretch. Prior work has shown that stretch has a significant impact on the sensitivity of elastic tissue to elastases (Burke et al. 2009). Stretch uncoils elastin fibers exposing hydrophobic binding and cleavage sites for elastase (Suki et al. 2012). A third limitation is that in vitro both the intimal (innermost) and adventitial (outermost) surfaces are treated and this could increase potency relative to the situation in vivo where only the adventitial surface is treated.

Currently, vonapanitase is in phase 3 human clinical trials (PATENCY‐1 and PATENCY‐2) in patients with CKD undergoing AVF creation (clinicaltrials.gov; NCT02110901 and NCT02414841). The goal of the clinical trials is to establish the safety profile of vonapanitase and determine if vonapanitase applied to the outflow vein at the time of AVF creation can improve the success and longevity of AVF surgery.

## Author Contributions

Burke, Bunton, Bingham, and Moss participated in research design. Bingham, Moss, and Starcher conducted the experiments. Burke, Wong, Bland, and Franano contributed to writing of the manuscript. Burke and Moss performed data analysis.

## Disclosure

None declared.
